# Gratitude predicts prosocial tendency through psychological resilience—cross-sectional study in Arab cultures

**DOI:** 10.3389/fpsyg.2024.1382093

**Published:** 2024-05-09

**Authors:** Ayman Abdullah Hazza Alamri, Mohammed Hasan Ali Al-Abyadh

**Affiliations:** ^1^Department of Special Education, College of Education in Al-Kharj, Prince Sattam bin Abdulaziz University, Al-Kharj, Saudi Arabia; ^2^College of Education, Thamar University, Dhamar, Yemen

**Keywords:** gratitude, prosocial tendency, psychological resilience, Saudi Arabia, Egypt

## Abstract

This study aimed to investigate the relationship between gratitude and prosocial tendency and the potential mediating role of psychological resilience in this association. Six hundred and seventy-six Saudi Arabia and Egyptian students completed the Gratitude Scale, Prosocial tendency Scale and Psychological Resilience Scale. The results showed no significant differences in gratitude, prosocial tendency and psychological resilience according to culture or gender in both countries. Moreover, gratitude positively correlated with prosocial tendency and psychological resilience. Furthermore, the results revealed that psychological resilience significantly mediated the association between gratitude and prosocial tendency. We conclude that gratitude predicts prosocial tendency and psychological resilience plays a mediating role in this association among Arab societies.

## Introduction

Gratitude, acknowledged as an intricate and multifaceted emotion (Algoe, 2012), has emerged as a focal point in psychological research owing to its profound influence on diverse dimensions of well-being (Wood et al., 2010). An intriguing facet of gratitude lies in its predictive capacity for prosocial tendency—an occurrence wherein individuals actively engage in actions that benefit others. This essay delves into the nuanced relationship between gratitude and prosocial tendency, with a specific emphasis on the mediating role of psychological resilience in this dynamic interplay.

Positioned as a cornerstone in fostering connections and fortifying social bonds, gratitude propels individuals toward elevated prosocial tendencys, encompassing acts of kindness, generosity, and empathy (Allen, 2018). This multifaceted concept unfolds as a predisposition to acknowledge and respond to benevolent gestures, reflecting an appreciation for the positive contributions individuals make to our lives.

The research underscores a robust connection between gratitude and prosocial tendency, indicating that experiencing gratitude inclines individuals toward actions that benefit others, ranging from simple acts of kindness to more profound altruistic behaviors. This connection highlights the potential motivational force of gratitude behind prosocial conduct, examined through various dimensions such as virtue, emotion, motive, moral feeling, coping reaction, attitude, and skill (Emmons and Crumpler, 2000).

Moreover, empirical evidence suggests that focusing on events for which one is grateful contributes to increased life satisfaction, optimism, and a host of other benefits (McCullough et al., 2002; Emmons and Paloutzian, 2003). These benefits extend to the formation of Prosocial tendency, enhanced well-being (Weinstein and Ryan, 2010), and personal fulfillment (Ellison, 1991).

Gratitude and psychological resilience emerge as complementary forces in a powerful synergy. Grateful individuals, by focusing on the positive aspects of their experiences, may enhance their psychological resilience. Simultaneously, those with high levels of resilience may find it easier to maintain a gratitude-oriented perspective, even in the face of challenges. This reciprocal relationship suggests that gratitude and psychological resilience reinforce each other, creating a robust foundation for prosocial tendencies.

Psychological resilience, recognized as an indispensable factor in overall well-being, has been explored as a potential mediator between gratitude and prosocial tendency. Recent studies link psychological resilience as a coping method to various psychological and mental health issues (Wu et al., 2020). It serves as a process involving reintegrating into normal functioning after a traumatic experience, thereby aiding individuals in coping with stress and reducing negative feelings (Hartley, 2011) while enabling them to handle challenging situations (Han et al., 2018).

In the context of Arab societies, where cultural norms significantly influence daily life, and psychological resilience seamlessly integrates into daily existence, understanding how gratitude correlates with prosocial tendency becomes paramount. This study aims to investigate the relationship between gratitude and prosocial tendency and the potential mediating role of psychological resilience in this association.

### Gratitude and prosocial tendency

Gratitude is conceptualized in various dimensions as a virtue, emotion, motive, moral feeling, coping reaction, attitude, and skill (Emmons and Crumpler, 2000). Gratitude, defined as the inclination to recognize and respond to the kindness of others, emerges as a valuable trait (McCullough et al., 2002). This emotional trait involves acknowledging and responding with gratitude to others’ contributions, even in adverse circumstances (Emmons and Mishra, 2011; Allen, 2018).

Research suggests that gratitude is linked to numerous benefits, including enhanced life satisfaction, improved mental well-being, and overall positive psychological outcomes (McCullough et al., 2002; Weinstein and Ryan, 2010). Gratitude is a powerful catalyst, to shape prosocial tendency. Acknowledging its role as a predictor, gratitude initiates a positive feedback loop that extends beyond individual well-being to influence social dynamics. Notably, expressing gratitude becomes a cornerstone in fostering connections and strengthening social bonds. This, in turn, leads to an elevation of prosocial tendencys, where grateful individuals exhibit kindness, generosity, and empathy (Allen, 2018). The reciprocity of positive interaction becomes evident as gratitude serves as a coping mechanism, allowing individuals to find silver linings in challenging situations and promoting a more optimistic outlook on life (Hill et al., 2013).

Prosocial activity, recognized as positive social conduct, takes center stage in this dynamic. Within the dimensions of general compliance and volunteering, considered crucial aspects of prosocial tendency (Ucho and Atime, 2013), a five-factor framework emerges. Cooperation, courtesy, competitiveness, and decision-making become the pillars of this prosocial tendency, each uniquely contributing to workplace performance. Several studies found a strong relationship between gratitude and prosocial tendency. For example, four studies conducted by Bartlett and DeSteno (2006) found strong evidence that gratitude played an important role in facilitating costly helping behavior in a manner distinct from that of a generally positive state or simple awareness of prosocial norms.

A recent study conducted by Pang et al. (2022) found that gratitude correlated to prosocial tendency. This study’s results indicated that perspective-taking, fantasy, empathic concern, and personal distress, respectively, played a mediating role between gratitude and prosocial tendency. Another study by Tian et al. (2015) investigated the role of prosocial tendency on the association of gratitude with subjective well-being among 706 Chinese elementary school students. The results found that gratitude positively correlated to prosocial tendency and prosocial tendency partially mediated the association of gratitude on school satisfaction and positive effect in school.

### Psychological resilience as a mediator

American Psychological Association defines psychological resilience as a mechanism of reasonable adjustment in the face of trauma, adversity, danger, tragedy, or other severe sources of stressors (Masten et al., 1990). The use of psychological resilience as a coping way has been linked to several psychological and mental health issues (Wu et al., 2020). According to Garg (2017), it is a process that involves reintegrating into normal functioning after a traumatic experience. Therefore, building psychological resilience among university students might help them cope with stressful situations and do better academically by reducing their negative feelings (Hartley, 2011), and allowing them to handle stressful situations (Han et al., 2018). It has been shown that encouraging resilience has favorable effects on lowering depression among university students (Zamirinejad et al., 2014) and on lowering mental health issues (such as depression symptoms, anxiety, and stress, etc) (Fenwick-Smith et al., 2018).

Resilience is therefore defined as positive results valued and helpful after enduring a problematic incident (Richardson and Waite, 2002). Psychological resilience is the capacity to recover from any challenging circumstances. As a result, determining the function of this capacity becomes much more critical. Research by Haroz et al. (2013) has demonstrated a favorable association between resilience and prosocial conduct among adolescents. and the presence of a statistically between psychological resilience and the effectiveness of the academic self (Al-Abyadh and Azim, 2020). Additionally Carelli et al. (2020) revealed that elevated levels of resilience play a crucial role in children’s social and emotional growth. This can potentially enhance their psychological capabilities and facilitate the development of prosocial tendency (Xue et al., 2022). A study conducted by Lasota et al. (2020) investigated the mediating role of resilience between empathy and gratitude among 214 adults. The results found that resilience mediated the association of empathy and gratitude. Another study by Listiyandini (2018) investigated the role of gratitude on psychological resilience among 200 adolescents in Jakarta and Bekasi and found that gratitude positively predicted psychological resilience.

In a recent investigation by Arnout and Almoied (2021), the interplay among gratitude, resilience, well-being, and counselors’ creativity was explored among a sample of 610 psychological counselors. Their findings unveiled a significant predictive relationship: gratitude emerged as a precursor to resilience, while resilience directly influenced creativity. This study underscores the intricate connection between gratitude and various facets of psychological well-being, including resilience.

Scholarly literature supports the notion that individuals who routinely experience and express gratitude tend to exhibit heightened levels of resilience, characterized by their adeptness in rebounding from adversity while maintaining psychological equilibrium (Wood et al., 2010). Additionally, psychological resilience has been associated with prosocial tendency, such as acts of kindness, empathy, and cooperation. Resilient individuals demonstrate a greater propensity to engage in altruistic actions, even amidst challenging circumstances (Ozbay et al., 2007).

Given these established associations, it is conceivable that resilience serves as a mediator between gratitude and prosocial tendency. That is to say, gratitude may cultivate resilience, which in turn empowers individuals to both desire and enact behaviors that benefit others. Chancellor and Lyubomirsky (2013) supported this notion in their study, where gratitude interventions not only heightened gratitude levels but also bolstered resilience among participants, suggesting a potential causal relationship.

Furthermore, empirical evidence provided by Jans-Beken et al. (2019) corroborates the proposed mediation model, demonstrating that psychological resilience acts as a mediator between gratitude and well-being outcomes. Building upon these findings, our current study seeks to explore the potential mediating role of psychological resilience in elucidating the relationship between gratitude and prosocial tendency.

### Cultural background of Saudi Arabia and Egypt

The present study examines data collected in Saudi Arabia and Egypt, two predominantly Muslim countries. Islam in these regions emphasizes social responsibility and helping others (Ibrahim, 1999). Collectivism, where the well-being of the group is valued alongside individual needs, is a prominent cultural characteristic (Hofstede, 2020).

Saudi Arabia, located in the Middle East, possesses a strong Islamic culture that emphasizes social harmony, respect for elders, and aiding those in need (Al-Kreathy and Hassan, 2018). However, modernization is fostering a rise in individualistic tendencies alongside collectivism (Househam, 2010). In contrast, Egypt, situated in North Africa, boasts a rich cultural heritage influenced by Pharaonic, Greek, Roman, and Arabic traditions. Egyptians value strong communities, social support networks (El-Gohary, 2008), and hospitality (Darwich and Saad, 2011).

Research on gratitude within Arab contexts is emerging (Al-Kreathy and Hassan, 2018). While the expression of gratitude might differ from Western cultures (e.g., focusing on thankfulness toward God) (Al-Kreathy and Hassan, 2018), it appears to be a relevant concept in Arab societies. There’s also a growing interest in understanding prosocial behavior in Arab countries (Mujtaba et al., 2019). Studies explore how social norms, religious beliefs, and in-group collectivism promote helping behaviors (Mujtaba et al., 2019). Additionally, research on psychological resilience in Arab populations is increasing (Asiri, 2011). Studies examine how cultural factors like religion, social support (El-Gohary, 2008), and coping mechanisms influence resilience in facing challenges (Asiri, 2011).

### The current study

Gratitude predicts prosocial tendency, yet its influence mediated by psychological resilience remains unclear. Therefore, this cross-sectional study aimed to investigate the relationship between gratitude and prosocial tendency and the potential mediating role of psychological resilience in this association in Saudi and Egypt students, so our hypotheses will be as follows:

*H1*: Gratitude positively correlates with prosocial tendency and psychological resilience.

*H2*: Psychological resilience plays a mediating role in the association of gratitude and prosocial tendency.

## Method

This study is designed as a cross-sectional study to test the relationship between gratitude and prosocial tendency and the potential mediating role of psychological resilience in this association in Saudi and Egypt samples.

### Participants and procedures

A total of 676 adults enrolled in universities in the Kingdom of Saudi Arabia and Egypt. As a result, a Google Docs online survey was created targeting undergraduate students representing Saudi and Egyptian Universities. After receiving formal clearances, we distributed the questionnaire to students via their registered emails at these universities. They received informed consent assuring the privacy of their responses. The sample age was 18–26 years (*M* = 22.44, SD =3.271). For more details, see Table 1.

**Table 1:** Demographics of the study participants.

Variable	Frequency	Percent%
Country		
Saudi	413	61.1%
Egypt	263	38.9%
Gender		
Male	395	58.4%
Female	281	41.6%

### Measures

#### Gratitude

The measurement of Gratitude which was created by McCullough et al. (2002) was used. The Arabic version has demonstrated reliability and validity in Arabic contexts (Azab, 2019). The Arabic version of the Gratitude Scale consists of 30 items, with respondents rating each item on a seven-point Likert-type scale ranging from 1 (*very untrue of me*) to 7 (*very true of me*). Example of items used in this scale is “*How often do you feel grateful for the good things in your life*?” The scale demonstrated good internal consistency, as indicated by a Cronbach’s alpha coefficient of 0.78.

#### Prosocial tendency

The Prosocial Tendency Scale which modified from Luengo Kanacri et al. (2021) was used in this study. The Arabic version of this scale has been proven to be reliable and valid in Arabic contexts (Salem, 2023). The scale consists of 16 items, and participants rated each item on a 5-point Likert-type scale ranging from 1 (*strongly disagree*) to 5 (*strongly agree*). Example of items used in this scale is “*I would go out of my way to help a stranger in need.*” The Cronbach’s alpha coefficient for the scale was calculated to be 0.83.

#### Psychological resilience

The Psychological Resilience Scale which developed by Windle et al. (2008) was used in our study. The Arabic version of this scale has been developed and proven to be reliable and valid in Arabic contexts (Zidane, 2021). The Arabic version consists of 29 items, and participants rated each item on a 5-point Likert-type scale ranging from 1 (*strongly disagree*) to 5 (*strongly agree*). Example of items used in this scale is “*Even during difficult times, I can usually find a way to cope.*” The Cronbach’s alpha coefficient for the scale was calculated to be 0.87.

### Data analysis

The data were analyzed in IBM SPSS v.23. To find the differences among study variables according to culture and gender, Independent samples T-Test was administered. To establish the relationship among study variables, Pearson correlation was calculated. For the mediation analysis by Hayes (2012), Process Macro was used. Furthermore, Model 59 of PROCESS macro was adopted (Hayes, 2012), for investigating the mediating effect. The results of the analyses are described in detail in the next section.

## Results

Before starting the final analysis, the full data set was initially checked for accuracy and univariate and multivariate outliers were discovered. The missing data information was obtained through frequency tabulation. By the requirements of the study, only cases with information on all scales were deemed complete (Table 2).

**Table 2:** Psychometric properties of the instruments.

	Variables		K	α	M	SD	Skew	Kurt
1	Gratitude	Saudi	31	0.88	28.90	5.17	−0.39	0.03
		Egypt	28	0.79	27.67	5.09	−0.38	0.02
2	Prosocial tendency	Saudi	20	0.74	29.20	5.02	−0.41	−0.01
		Egypt	15	0.71	27.86	4.90	−0.40	−0.01
3	Psychological resilience	Saudi	28	0.86	28.12	6.24	−0.23	−0.51
		Egypt	26	0.77	26.90	5.90	−0.22	−0.49

### Correlations among study variables

To study the relationship among study variables, Pearson correlation was calculated. The results (Table 3) showed that gratitude positively correlated with prosocial tendency and psychological resilience.

**Table 3:** Correlations among study variables.

Variables		1	2	3
Country		Saudi		
Gratitude	Egypt	1	791	619
Prosocial tendency		0.812	1	489
Psychological resilience		0.528	0.445	1

### Mediating role of psychological resilience

**Figure 1:**
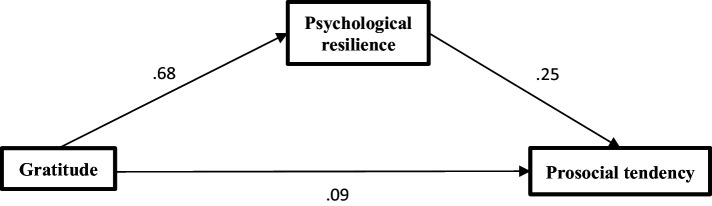
The mediating role of psychological resilience among Saudi sample.

**Figure 2:**
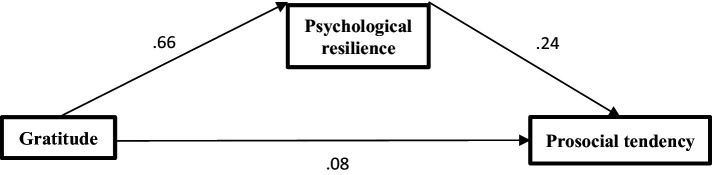
The mediating role of psychological resilience among Egyptian sample.

This study uses the PROCESS program to investigate the mediating role of psychological resilience on the association of gratitude with prosocial tendency (Hayes, 2012). It adopts the bias-corrected percentile bootstrap method (model 4, sample = 5,000, 95% CI) to test the mediating effect results. When Gratitude was used as the independent variable, Prosocial tendency as the dependent variable, and psychological resilience as the mediating variable, the results were as follows: The total effects of gratitude on prosocial tendency is significant (Saudi: *β* = 0.26, *p* < 0.01; Egypt: *β* = 0.23, *p* < 0.01) the direct effects of gratitude on prosocial tendency (Saudi: *β* = 0.09, *p* < 0.01; Egypt: *β* = 0.08, *p* < 0.01). Gratitude positively predicted prosocial tendency, and psychological resilience positively predicted prosocial tendency (see Table 4). Finally, the bias-corrected percentile bootstrap method indicated that the indirect effect of gratitude on prosocial tendency through psychological resilience was significant (Saudi: *β* =0.25, SE = 0.04, 95% CI = [0.17, 0.32]; Egypt: *β* =0.24, SE = 0.04, 95% CI = [0.16, 0.31]). This indicates that the effects of gratitude on prosocial tendency were partially mediated by psychological resilience (Figures 1, 2).

**Table 4:** The mediating role of psychological resilience.

Predictors		Psychological resilience			Prosocial tendency			Psychological resilience-Prosocial tendency		
		Β	SE	T	Β	SE	T	Β	SE	T
Gratitude	Saudi	0.68	0.03	18.03	0.09	0.02	3.89	0.25	0.02	10.14
	Egypt	0.66	0.03	16.70	0.08	0.02	3.43	0.24	0.02	9.78

## Discussion

The study aims to investigate the relationship between gratitude and prosocial tendency and the potential mediating role of psychological resilience in this association. Our results also found that gratitude positively correlated with prosocial tendency and psychological resilience; that indicates that cultivating gratitude may have broader implications for individual well-being and social dynamics. It could serve as a positive force that not only influences how individuals interact with others but also contributes to their ability to adapt and thrive in the face of life’s ups and downs. This connection between gratitude, prosocial tendency, and psychological resilience highlights the multifaceted nature of positive psychological traits and their impact on human behavior and resilience. These results consent with studies such as (Bartlett and DeSteno, 2006; Tian et al., 2015; Peng et al., 2020). Finally, these correlations between gratitude and both prosocial tendency and psychological resilience are a little stronger in Saudi Arabia compared to Egypt. However, it’s not our aim to investigate these differences.

In addition, our study results also found a significant positive association between gratitude and prosocial tendency in both Saudi Arabia and Egypt. People who report feeling more grateful are also more likely to engage in helpful behaviors toward others. Results indicated that psychological resilience partially mediates the relationship between gratitude and prosocial tendency. This means that gratitude’s positive influence on helpfulness is partly explained by its effect on psychological resilience. People who feel grateful tend to develop greater resilience, which in turn leads to more prosocial behavior. This also indicates that individuals who experience gratitude are more likely to engage in prosocial tendency. The fact that psychological resilience partially mediates this relationship implies that individuals with higher levels of psychological resilience may be better equipped to translate their feelings of gratitude into prosocial actions. Resilience could act as a psychological mechanism that enables individuals to overcome obstacles and still engage in positive behaviors toward others (Davydov et al., 2010; Fletcher and Sarkar, 2013).

Therefore, gratitude involves recognizing and appreciating the positive aspects of one’s life, which can contribute to building psychological resilience; where individuals who practiced gratitude showed improved psychological well-being and resilience (Panhwar and Malik, 2023). On the other hand, Psychological resilience refers to the ability to bounce back from adversity, cope with challenges, and maintain well-being in the face of stress. Individuals with higher levels of psychological resilience are often better equipped to engage in prosocial tendency (Haroz et al., 2013). Resilient individuals may have greater empathy, compassion, and a sense of social responsibility, which are essential components of prosocial tendency (Lim and DeSteno, 2016).

### Limitations and future research direction

The study may have limitations, first in terms of generalizability, as it focused specifically on students in Saudi Arabia and Egypt. Extrapolating these findings to other age groups, professions, or cultural contexts may not be warranted without further research. Second, the reliance on self-reported measures for variables such as gratitude, psychological resilience, and prosocial tendency introduces the possibility of response bias. Participants may provide socially desirable responses, affecting the accuracy of the data. Third, while the research identifies associations between gratitude, psychological resilience, and prosocial tendency, it does not establish causation. Future studies employing experimental designs or interventions could provide more robust evidence of causal relationships. Fourth, the study may not fully capture the cultural nuances that influence the interpretation of gratitude, psychological resilience, and prosocial tendency. Future research should employ culturally sensitive measures and qualitative methods to gain a deeper understanding.

Future studies should replicate the research with diverse samples across various cultures and demographics to validate and extend the findings. This could enhance the generalizability of the proposed mediation path. Conducting longitudinal studies would allow researchers to explore how gratitude, psychological resilience, and prosocial tendency evolve. This approach would provide insights into the dynamic nature of these constructs. In addition, implementing interventions to enhance gratitude or psychological resilience and observing their effects on prosocial tendency would help establish causation. Such interventions could inform practical strategies for promoting prosocial tendency. Finally, conducting more in-depth, context-specific cultural studies within Saudi Arabia and Egypt could uncover variations in the relationships among gratitude, psychological resilience, and prosocial tendency across different regions or communities.

## Data availability statement

The data analyzed in this study is subject to the following licenses/restrictions: we can provide the data when it requested. Requests to access these datasets should be directed to alabyd62@gmail.com.

## Ethics statement

Ethical approval was not required for the studies involving humans because its a cross-sectional study, not experimental work. The studies were conducted in accordance with the local legislation and institutional requirements. The participants provided their written informed consent to participate in this study.

## Author contributions

MA-A: Data curation, Formal analysis, Funding acquisition, Methodology, Writing – original draft, Writing – review & editing. AA: Investigation, Project administration, Supervision, Writing – review & editing.
